# Effects of Prolonged Sitting with Slumped Posture on Trunk Muscular Fatigue in Adolescents with and without Chronic Lower Back Pain

**DOI:** 10.3390/medicina57010003

**Published:** 2020-12-23

**Authors:** Kyoung-Sim Jung, Jin-Hwa Jung, Tae-Sung In, Hwi-Young Cho

**Affiliations:** 1 Department of Physical Therapy, Gimcheon University, Gimcheon 39528, Korea; 20190022@gimcheon.ac.kr; 2 Department of Occupational Therapy, Semyung University, Jecheon 27136, Korea; otsalt@semyung.ac.kr; 3 Department of Physical Therapy, Gachon University, Incheon 21936, Korea

**Keywords:** lower back pain (LBP), sitting, fatigue, discomfort

## Abstract

*Background and Objectives:* This study investigated the effects of prolonged sitting on trunk muscular fatigue and discomfort in participants with and without chronic lower back pain (LBP). *Material and Methods:* This study included 15 patients with LBP and 15 healthy controls. All participants were instructed to sit on a height-adjustable chair with their knee and hip joints bent at 90° for 30 min, in slumped sitting postures. Surface electromyography was used to assess the median frequency of the internal obliques (IO)/transversus abdominis (TrA) and multifidus (MF) muscles. Perceived discomfort was measured using a Borg category ratio-scale. Median frequency of the trunk muscles and perceived discomfort after 30 min of sitting were compared with baseline. *Result:* There were no significant differences within the group and between both groups in the median frequency of bilateral IO and MF muscles. The LBP group showed significantly greater perceived discomfort after prolonged sitting, as compared to the control group. *Conclusions:* Prolonged sitting with slumped posture could increase the risk of experiencing lower back discomfort.

## 1. Introduction

Sitting for a long time can cause discomfort in the lower back [1], and maintaining the sitting posture for extended periods is reportedly associated with the endurance of lower back muscles [2]. By analyzing the sitting posture for 1 h in healthy individuals and those with lower back pain (LBP), it was determined that participants in both groups adopted a slumped sitting posture after 20 min, and that LBP patients showed an asymmetrical pattern of gluteal pressure.

A reduction in back muscle endurance could be an independent predictor of back pain [3,4]. Individuals with chronic LBP have a higher pain intensity and lower tolerance to low-intensity static loads, as compared to healthy individuals, when sitting for long periods [5]. Additionally, patients with LBP show a decrease in trunk motor variability [6,7] and their back muscles are more easily fatigued [8].

In recent years, due to the flexibility of mobile devices, people are increasingly using portable equipment without a desk [9]. In unsupported sitting conditions without a backrest or desk, people often adopt poor sitting postures that increase the risk of musculoskeletal disorders [10,11]. Although some epidemiological studies and systematic reviews determined that sitting time and posture are not significantly associated with the development of LBP [2,12,13], other studies showed that sitting in a bad posture for a long period increases LBP and lumbar discomfort [2,14,15,16].

Sitting posture can affect the trunk muscle activity [17], and different muscles are predominantly stimulated, depending on the sitting posture [18]. Studies comparing the activity of trunk muscles based on posture reported that slumped sitting significantly decreased the activity of the internal obliques (IO)/transversus abdominis (TrA), when compared to upright sitting [17,19]. In recent years, studies indicated that muscular fatigue is present in the trunk muscles of healthy individuals who maintain a slumped sitting posture for a long period [9,20]. Ringheim et al. [5] also found that there was no significant difference between LBP patients and healthy individuals, when comparing muscular fatigue after prolonged sitting with a forward trunk inclination. There are many studies on muscular fatigue in patients with LBP, but most of them measured muscular fatigue on isometric trunk extension in the prone or sitting postures. Only a few studies analyzed the muscular fatigue associated with long periods of sitting posture [9,20]. In fact, no study investigated the changes in muscles during prolonged slumped sitting posture, especially in LBP patients.

Therefore, this study aimed to identify fatigue and discomfort of the trunk muscles in individuals with and without non-specific chronic LBP, when using a mobile device in a slumped sitting posture for 30 min.

## 2. Materials and Methods

### 2.1. Participants

This study included 15 patients with LBP and 15 healthy individuals. The inclusion criteria were LBP experienced for ≥3 months, aged between 10–19 years, pain intensity ≥3 on the visual analogue scale. Participants in the control group had no previous LBP history. The exclusion criteria were (1) using other treatments or drugs to manage LBP, (2) presence of musculoskeletal problems other than LBP, (3) history of surgery related to LBP, (4) history of neurological system disorders, and (5) body mass index (BMI) > 30 (kg/m^2^), due to potential difficulty in obtaining surface electromyography (EMG) measures [9]. 

### 2.2. Protocol

In this study, participants were seated on a chair without desks or backrests to perform a 30-min mobile-device usage task. Before conducting the measurement, the height of the chair was adjusted for each participant to bend the knees at 90°. To represent the continuous static sitting posture while using the mobile device without support [21,22,23], they were asked to maintain a relaxed kyphotic posture, without any significant body movements, and place their hands holding the mobile device on their knees. To minimize any postural stress, the participants were allowed to switch to a more upright sitting posture. All participants were asked to perform the typing tasks at their normal speed. The perceived discomfort level was rated for each participant at the start and completion of the task. Furthermore, EMG data were constantly recorded during the 30-min task period to measure muscular fatigue.

### 2.3. Outcome Measurements

A four-channel radio EMG device (TeleMyo T1500, Noraxon Inc., Arizona, USA) was used to measure the muscular fatigue. Electrodes were attached to the bilateral IO/TrA and multifidus (MF) muscles. The electrodes were attached 1 cm medial to the anterior superior iliac spine for IO/TrA and 2 cm lateral to the L5 spinous process for MF. The sampling frequency was set to 1000 Hz and a bandwidth of EMG recordings was set between 20–450 Hz. After a full-wave rectification, the EMG signals were processed with a root-mean-square smoothing algorithm. To minimize skin resistance, any hair on the target muscle was removed, and the skin was wiped using alcohol swabs, before attaching the electrodes.

The median frequency was obtained from the EMG data analysis of the first and last 5 min. The mean value of the EMG median frequency for the 5-min periods was calculated and normalized to the initial value. The median frequency change was used as the muscle fatigue index [9].

The Borg category-ratio (CR-10) scale was used to measure the perceived discomfort. The Borg CR-10 measured the degree of discomfort in the lower back on a 10-point scale. This scale indicated that higher scores indicate greater discomfort [24].

### 2.4. Data Analysis

Statistical Package for the Social Sciences version 21.0 (IBM-SPSS Inc., Chicago, IL, USA) was used for statistical data analysis. The Shapiro-Wilk test was used to assess the normality of variables. The chi-square test for categorical variables and the independent *t*-test for continuous variables were used to analyze and compare the general characteristics of the participants of the LBP and the control groups. The paired *t*-test was used for within-group comparisons and the independent *t*-test for the between-group comparisons. The level of statistical significance was set at 0.05. 

## 3. Results

### 3.1. General Characteristics of Participants

We calculated the sample size based on previous studies. The effect size was set to 0.95 and the alpha level was set to 0.05, respectively. Additionally, the power was set to 80%. It was calculated that 14 people were needed for each group, and we recruited 15 subjects per group considering drop-out. Table 1 shows the characteristics of participants in both groups. There was no significant difference in any characteristics.

### 3.2. Comparison of Median Frequency

**Figure 1:**
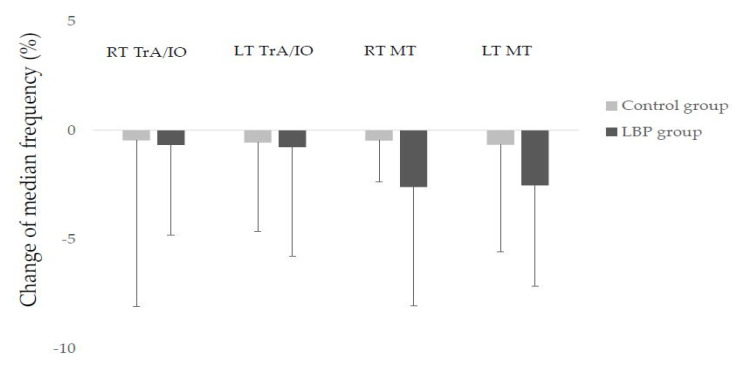
Mean change of median frequency in trunk muscles after prolonged sitting with slumped posture. EMG median frequency was normalized to the initial value.

There was no significant difference in the median frequency of IO, before and after sitting in both groups. In the case of MF, the median frequency tended to decrease in the LBP group, but it was not statistically significant (Table 2, Figure 1).

### 3.3. Comparison of Body Perceived Discomfort

**Figure 2:**
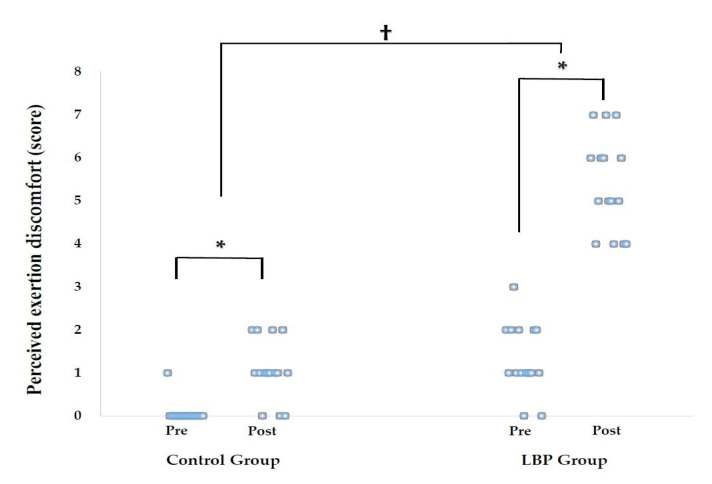
Mean of perceived discomfort after prolonged sitting with slumped posture. * Significant differences between pre and posttest (*p* < 0.05). † Significant differences between the group (*p* < 0.05).

After sitting for 30 min, body discomfort was significantly increased in the both groups (*p* < 0.001) and there were significant differences between groups (*p* < 0.001) (Table 3, Figure 2).

## 4. Discussion

Sitting for prolonged period results in posture changes such as the flatness of the lumbar-lordotic curve [25] and chronic muscle deconditioning that decreases muscle activity [26]. This can cause muscular fatigue even at low loads, when adopting static posture for a long period [27].

In this study, we measured the muscular fatigue in the IO/TrA and MF muscles, when individuals with and without LBP used mobile devices in a slumped posture for 30 min. The IO/TrA and MF are representative local systems for increasing lumbar stability and balancing compressive forces on the upper lumbar segment of the spine [28]. A study comparing the fatigue of trunk muscles in an upright, slumped, and forward leaning posture for 1 h in office workers reported IO/TrA fatigue only in the slumped sitting posture [5]. Another study measuring the fatigue of trunk muscles in healthy subjects when using a laptop with unsupported sitting and slumped posture for 40 min, showed fatigue of trunk muscles such as the MF, IO, erector spinae, and external obliques. This suggested that these muscles were continuously activated to stabilize the trunk as postural control muscles, while performing tasks [9]. According to a study that investigated telecommuting and musculoskeletal problems in the mobile worker population, poor posture aggravated musculoskeletal pain, due to the use of non-ergonomic equipment that cannot be controlled when working from home [29]. Adolescents spend a lot of time at school. In the case of school, there are regular breaks, but since it is an environment where adjustable desks and chairs cannot be used like those of home workers, low back pain and muscle fatigue might increase due to uncomfortable posture for a long time. In this study, after prolonged sitting with slumped posture, there was no significant change in the median frequency of the IO in either group. In this study, the mean frequency of MF was not statistically significant, but it only tended to decrease in the low back pain group. A study comparing the trunk muscle activity in different postures reported that the activity of trunk muscles, including IO [17,30] and MF [31], was lower in a slumped sitting posture than in an erect sitting posture, due to passive structure support. This resulted in the lowest risk of muscular fatigue [31,32]. However, the study also reported an increase in MF activity in patients with LBP, compared to healthy participants, which could be the muscle’s protective response to pain in the slumped sitting posture [33]. Most patients with LBP who participated in this study reported pain during trunk flexion. Therefore, it is thought that a slumped sitting posture increased MF activity in LBP patients and affected muscle fatigue over time.

LBP patients have decreased muscle activity variation, which means that despite postural changes, it is difficult to deactivate the lumbar muscles, thus causing local muscular fatigue even at low levels of isometric muscle contraction [5]. Indeed, it is difficult for patients with pain to relax their muscles after muscle activation, and rest periods are reduced during repetitive routine tasks [34]. Ringheim et al. [5] also found that muscle activity variability was decreased and pain intensity was increased in LBP patients, as compared to healthy participants, after sitting for 30 min with a forward leaning posture. However, there was no significant difference in median frequency between both the groups.

The local muscular fatigue of the lumbosacral region due to continuous sitting, which appears mainly due to a decrease in mean power or median frequency, can be measured by EMG [35]. The feasibility of detecting muscular fatigue when measuring for >30 min was demonstrated even during low-level contraction [36,37]. However, EMG-based assessments of muscular fatigue had a lower impact when the activity was <15% of maximal muscle capacity [38,39]. In standing or sitting postures, the trunk muscles maintained levels <10% of maximum activation [40,41]. In this study, the fatigue of the trunk muscles might not have been elicited due to the small activity of the measured posture and the relatively short measurement time for generating changes in median frequency. The large variation between the participants might be attributed to a reduced sensitivity of the surface EMG in measuring deep muscular fatigue, due to the complex, layered structure of the trunk muscles [18,41,42].

Additionally, this study measured the change in discomfort after slumped sitting, and found a significant difference between the groups. In the study conducted by Baker et al. [28], muscular fatigue did not change significantly after 2 h of sitting, but the discomfort in the lumbar region significantly increased due to the increase in the stress of passive tissues with decreasing lumbar lordosis. The slumped sitting posture was maintained against gravity with the spine flexed in the mid to end range, and the passive tissue took the load of the upper body [43,44]. Although not measured in this study, it was expected that the stress of the posterior spinal passive structure was greater in the LBP group than in the control group, as the LBP group maintained a kyphotic posture, while performing tasks.

This study compared the fatigue in the trunk muscles and discomfort of the lumbar region when individuals with and without LBP worked in a slumped sitting posture. The results showed no significant difference in muscular fatigue between the groups, but the discomfort was significantly higher in the LBP group. However, due to the small number of participants in this study, it would be difficult to generalize these findings. Furthermore, because of the short measurement time, the effect of LBP and sitting posture on muscular fatigue was not significant. It is necessary to measure muscular fatigue using various methods, such as motor variability and stimulation response, other than median frequency. Moreover, there is a need to study the changes in the spinal curvature of individuals, with and without LBP, when sitting for long periods.

## 5. Conclusions

Our results suggest that when sitting in a slumped position for a long time, the discomfort in the lower back increases regardless of muscle fatigue, and adolescent patients with LBP are more affected by these postures.

## Figures and Tables

**Table 1:** General characteristics of participants (*N* = 30).

Variables	LBP Group (*n* = 15)	Control Group (*n* = 15)	*p*
Sex (Male/Female)	11/4	10/5	0.690 ^a^
Age (years)	17.40 ± 2.85	17.53 ± 2.47	0.381 ^b^
Height (cm)	171.87 ± 5.96	168.53 ± 8.94	0.239 ^b^
Weight (kg)	68.87 ± 10.58	66.20 ± 13.02	0.543 ^b^
Postures that make symptoms worse (lumbar flexion/extension)	(13/2)		

Values are expressed as mean ± standard deviation. ^a^ chi-square test, ^b^ independent *t*-test; LBP: lower back pain.

**Table 2:** Median frequency of the subject before and after 30 min of sitting.

	Control Group		Difference	LBP Group		Difference	*p*
	Pre	Post		Pre	Post		
RT IO	100 ± 0.00	99.54 ± 7.60	−0.46 ± 7.60	100 ± 0.00	99.33 ± 4.13	−0.67 ± 4.13	0.927
LT IO	100 ± 0.00	99.44 ± 4.07	−0.56 ± 4.07	100 ± 0.00	99.23 ± 5.01	−0.77 ± 5.01	0.898
RT MT	100 ± 0.00	99.53 ± 1.88	−0.47 ± 1.88	100 ± 0.00	97.40 ± 5.42	−2.60 ± 5.42	0.161
LT MT	100 ± 0.00	99.34 ± 4.91	−0.66 ± 4.91	100 ± 0.00	97.48 ± 4.61	−2.52 ± 4.61	0.293

RTIO, Right internal oblique, LTIO, Left internal oblique, RTMT, Right multifidus, and LTMT, Left multifidus.

**Table 3:** Subject scores of disability before and after 30 min of sitting.

	Control Group		Difference	LBP Group		Difference	*p*
	Pre	Post		Pre	Post		
Disability	0.07 ± 0.26	1.07 ± 0.70	1.00 ± 0.65 *	1.33 ± 0.82	5.40 ± 1.12	4.07 ± 1.33 *	0.000

* Significant differences between pre and posttest (*p* < 0.05).

## Data Availability

The data of this study are available from the corresponding author upon reasonable request.
